# The effects of art therapy on anxiety, depression, and quality of life in adults with cancer: a systematic literature review

**DOI:** 10.1007/s00520-020-05869-0

**Published:** 2020-11-13

**Authors:** J. T. Bosman, Z. M. Bood, M. Scherer-Rath, H. Dörr, N. Christophe, M. A. G. Sprangers, H. W. M. van Laarhoven

**Affiliations:** 1 Department of Medical Oncology, Cancer Center Amsterdam, Amsterdam University Medical Centers, University of Amsterdam, Meibergdreef 9; D3-312, 1105 AZ Amsterdam, The Netherlands; 2 Faculty of Philosophy, Theology and Religious Studies, Radboud University–Nijmegen, Nijmegen, The Netherlands; 3 University of the Art Utrecht, Utrecht, The Netherlands; 4 Department of Medical Psychology, Cancer Center Amsterdam, Amsterdam University Medical Centers, University of Amsterdam, Amsterdam, The Netherlands

**Keywords:** Cancer, Oncology, Patients, Quality of life, Art therapy

## Abstract

**Purpose:**

While there is increasing evidence for the effectiveness of psychosocial support programs for cancer patients, little attention has been paid to creativity or art as a way of addressing their psychological problems and improving quality of life. This review provides an overview of interventional studies that investigate the effects of art therapy interventions on anxiety, depression, and quality of life in adults with cancer.

**Methods:**

We conducted a literature review with a systematic search. The databases PubMed/MEDLINE, PsycINFO, and EMBASE were searched for articles on art therapy among adult (18 years and above) cancer patients, published between September 2009 up to September 2019. Search terms were established for each database specifically. A total of 731 publications was assessed for relevance by title and abstract. The remaining 496 articles were examined using three inclusion criteria: interventions were guided by an artist or art therapist, participants were actively involved in the creative process, and anxiety, depression, and/or quality of life were included as outcome measures. Methodological quality of the included studies was appraised using specific checklists.

**Results:**

Seven papers met the inclusion criteria. Data was extracted from three non-randomized intervention studies and four randomized controlled trials. All studies used a quantitative design with validated outcome measures. Four articles described positive effects of art therapy on anxiety, depression, or quality of life in adults with cancer.

**Conclusion:**

Art therapy could possibly help decrease symptoms of anxiety and depression, and improve quality of life in adult cancer patients. However, because of the heterogeneity of the interventions and limited methodological quality of the studies, further research using stringent methods is needed.

**Supplementary Information:**

The online version contains supplementary material available at 10.1007/s00520-020-05869-0.

## Introduction

### Background

Receiving the diagnosis cancer may evoke strong emotions of anger and anxiety and can be considered traumatic [1]. When the emotional burden of being seriously ill stretches beyond patients’ ability to cope, it may even result in mental disorders [2]. Indeed, anxiety and depression disorders are common among cancer patients [3, 4], as they affect 10% and 20% of the cancer population respectively, which is two to three times higher compared to the general population [5]. Symptoms that are clinically relevant, but do not meet the DSM-criteria for an anxiety or depressive disorder, such as insomnia or distractibility, are even more frequent among cancer patients [6, 7]. These symptoms of depression and anxiety affect quality of life (QoL) adversely [8, 9].

The relevance of interventions that address psychological symptoms is increasingly recognized [10], and several supportive care interventions have been shown to be effective among cancer patients [11–13]. An example of such a supportive intervention is art therapy. Several definitions of art therapy are available, which are partly non-overlapping. The British Association of Art Therapists (BAAT) defined art therapy as “a form of psychotherapy that uses art media as its primary mode of communication” [14]. Similarly, art therapy is seen by Pamela et al. as a form of psychotherapy, practiced by trained art therapists, aiming at therapeutic goals [15]. Rather than an approach to enhance self-expression, others emphasize the creative process in art therapy that has healing effects and enhances patients’ well-being [16, 17].

A forensic psychiatry study showed the beneficial use of art therapy in the treatment of destructive aggression [18]. Haeyen et al. [19] found improvements in self-expression among patients with personality disorders undergoing art therapy. Another study highlighted the value of art therapy programs on emotion regulation in active duty military service members with post-traumatic stress disorder and traumatic brain injury [20].

In oncology, however, art therapy as a supportive care intervention is a relatively new and previous literature studies in this field contain some limitations. For instance, Geue et al. [30] and Wood et al. [31] use a variety of study designs, making it hard to draw conclusions because of the heterogeneity of the studies included. Furthermore, Ennis et al. [33] focus on the beneficial effects only, thus, not paying attention to potential negative outcomes. Most importantly, all reviews indicate that more research is needed in this field and since upcoming literature about art therapy in cancer care is increasing rapidly, more reviews may be relevant. Therefore, the present review provides a systematic literature overview of the available effectiveness of this form of therapy in adult cancer patients.

In this review, we will define art therapy as an art intervention, aimed at decreasing symptoms of anxiety, depression, and/or increasing QoL, which is delivered by someone with expertise in arts (an artist or professional art therapist). This ensures that there is professional guidance in the use and making of the art, although this does not necessarily involve professional psychotherapeutic involvement. An art therapy intervention may include all sorts of disciplines, like singing, drawing, painting, coloring, sculpting, writing, or poetizing. Our aim is to provide an overview of interventional studies that investigate the effects of art therapy interventions on anxiety, depression, and quality of life in adults with cancer. We focus on the *making* of art and will leave out passive forms, such as listening to music or looking at paintings.

## Methods

### Systematic literature review

We conducted a literature review with a systematic search to provide a summary of the evidence on the use of art therapy in cancer care.

### Search strategy and inclusion criteria

We performed a search in the databases PubMed/MEDLINE, PsycINFO, and EMBASE, because we deemed these to be most relevant to our research topic. We searched for publications from September 2009 up to September 2019 and no restrictions regarding publication type were made at this stage. We only included publications that were available in English. The search contained the following search terms: “art therapy” OR “art-making” AND “cancer” OR “oncology”. Search terms were adjusted for each database specifically and can be found in Supplementary Material. Using this strategy, we obtained a total of 968 articles. Of this entire search, 280 publications were derived from PubMed/MEDLINE: 256 from PsycINFO and 432 from EMBASE. Subsequently, 237 duplicate articles were removed. Two reviewers (HB and ZB) first screened the remaining articles by title and excluded clearly irrelevant articles. The remaining 496 publications were assessed by HB and ZB based on their title and abstract, using the following inclusion criteria: Studies including adults above the age of eighteen with cancer who were involved in art making in the presence of an artist or art therapist, employing anxiety, depression, and/or QoL as outcomes. To increase the validity of our results, we only included prospective cohort studies with a controlled design.

### Critical appraisal

The Critical Appraisal Tools by the Joanna Briggs Institute (JBI) were used to examine the methodological quality of the studies [21]. For the non-randomized intervention studies, we used the JBI Critical Appraisal Checklist for Quasi-Experimental Studies and for the randomized controlled studies we used the JBI Critical Appraisal Checklist for Randomized Controlled Trials.

### Data extraction and analysis

The following information was extracted from each study paper: authors, year, study design, number of patients, female to male ratio, number of patients in the intervention/control group, cancer diagnosis, duration and methods of the art therapy intervention, type of instructor of the art therapy intervention, outcome measures, and main findings. A descriptive analysis was performed to evaluate the results.

## Results

### Overview of articles

**Fig. 1:**
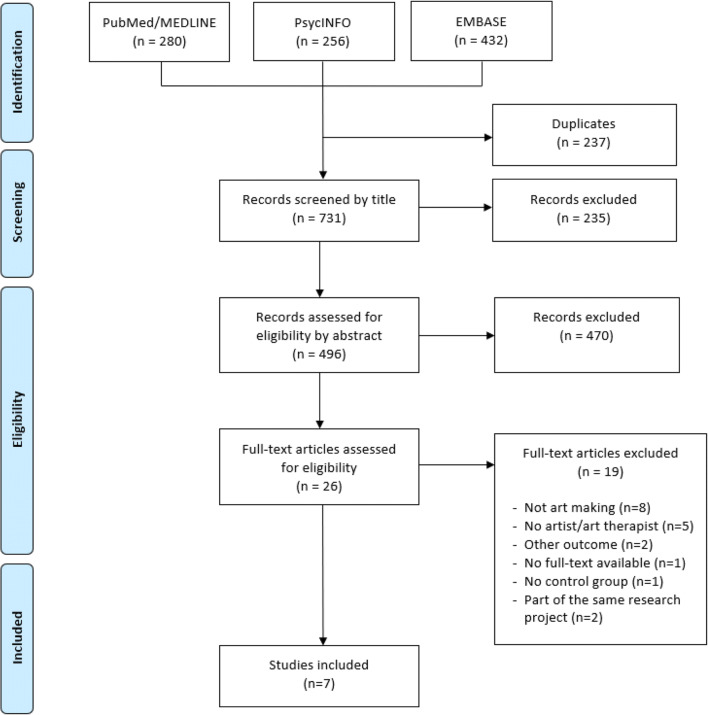
Selection strategy

Twenty-six articles were read in full text of which nineteen articles were excluded because they did not meet the inclusion criteria, for instance, they did not focus on art making or did not include an artist or art therapist. Hence, seven articles were suitable for further analysis. These included three non-randomized intervention studies and four randomized controlled trials. An overview of our selection strategy can be found in Fig. 1.

The number of participants reported in the articles varied between 24 and 183. Six out of seven papers included more than 50 patients. Three studies focused on female cancer patients only [22–24]. In the remaining articles, patents with a variety of cancer diagnoses were included. In general, more women than men participated in the art therapy trials. A complete overview of the sociodemographic characteristics was given in all studies, except for Radl et al. [24] who only reported age and race of the participants. Four articles described the diagnosis of the patients and their clinical characteristics [24–27].

All studies used a quantitative design with validated outcome measures. The Hospital Anxiety and Depression Scale (HADS) and the EORTC-QLQ C-30 were used most frequently as outcome measures. One study added a qualitative questionnaire to explore the satisfaction with the art therapy intervention [26].

### Critical appraisal

The non-randomized controlled trials were assessed based on nine questions about the methodological quality of the studies (Table 1). In all articles, the examined causes and effects were clear. The measurements were psychometrically robust and were applied both before and after the interventions. However, the patients in the control group were only similar to the patients in the intervention group in one study [26]. For example, in one study, the control group consisted of patients who declined participation in the art therapy program, which may have caused selection bias [25]. Also, it was often unclear whether the control group and the intervention group received similar cancer treatment apart from the art therapy intervention [25, 27].

**Table 1:**
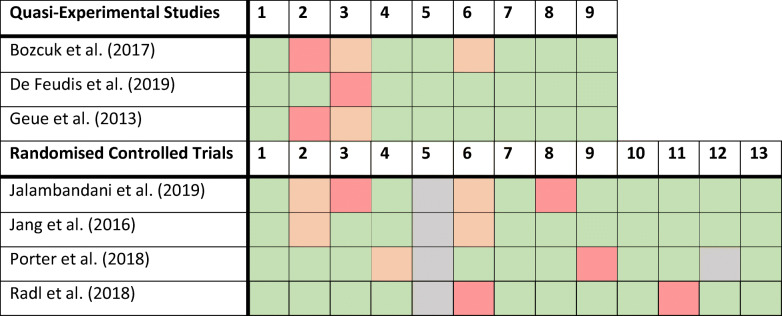
JBI critical appraisal^1^

^1^Green, answer is “Yes;” Orange, answer is “Unclear;” Red, answer is “No;” Blue, not applicable

The checklist regarding randomized controlled trials consisted of twelve questions. In all studies, true randomization was used; however, blinding the treatment was self-evidently not applicable in any of the studies [22, 23]. Porter and McConnell [28] noted that their outcome assessors were blinded. All seven articles used appropriate statistical analysis. Full elaboration of the answers to the questions on the checklists can be found in Supplementary Material. No studies were excluded based on their methodological quality.

### Description of the included articles

#### Bozcuk et al. [25]

Bozcuk and Ozcan [25] included participants from the outpatient chemotherapy unit Akdeniz University Medical Faculty in Antalya, Turkey. Patients were classified based on their previous exposure to painting art therapy and were divided into two intervention groups. Patients declining participation served as control group. An art therapist with experience in painting art therapy worked with everyone individually. First, he provided information about the materials and techniques and then let the patients make as much watercolor paintings as they wanted during a chemotherapy appointment. Afterward, the art therapist encouraged the patients to elaborate on the meaning and subject of their finished work. The number of finished paintings was registered as a representation of motivation.

#### De Feudis et al. [26]

De Feudis and Graziano [26] provided art therapy sessions of 90 min in the Medical Oncology Out-Patient unit of San Paolo, serving a population of adult cancer patients from Puglia, Italy. Each patient participated in one group session. The control group was on a waiting list to receive art therapy and meanwhile received usual care. A psychotherapist skilled in art therapy guided the sessions, assisted by a psycho-oncology team. The intervention took place in a room equipped with a large amount of art materials and background music. Groups consisted of a maximum of eight people, varying in age, gender, and diagnosis. The therapy focused on three principles: production of spontaneous artwork, provocation of self-reflection, and sharing experiences with group members. Afterward, all patients were offered the opportunity of additional psychosocial support.

#### Geue et al. [27]

The hemato-oncological patients in the study of Geue and Richter [27] were recruited from the Leipzig University Hospital, Germany. Hemato-oncological patients who lived too far away to participate formed the control group. Twenty-two weekly sessions of 90 min were held under the supervision of an art therapist. The groups included patients of different gender and age. The intervention consisted of three phases: becoming familiar with drawing, assisted by an artist, watercolor painting by oneself, and creating an individual book to express feelings. All decisions regarding the content or design of the book were made by patients themselves.

#### Jalambadani et al. [22]

Jalambadani and Borji [22] investigated Neyshabur women with breast cancer visiting the Razavi Hospital of Mashhad City, Iran. They conducted twelve weekly mindfulness-based art therapy (MBAT) sessions, lasting on average 90 min. The control group was on a waiting list to receive art therapy and was provided with usual cancer care. The MBAT-program focused on the procedure first used in Monti and Peterson [29], involving an introduction to art-making, self-picture assessment tasks, exploration of art materials and mind-body relationship, creative problem-solving, meditation, free art-making, and group discussions. The interventions were guided by an artist with psycho-oncological training.

#### Jang et al. [23]

Jang and Kang [23] examined the effects of mindfulness-based art therapy (MBAT) in women with breast cancer, who had received surgery and radiation therapy at Wonkwang University Hospital, South-Korea. The patients in the MBAT-group were provided with twelve weekly sessions lasting 45 min each. The qualified art therapist encourages the patients to express their inner feelings. Both the intervention group and the control group continued to have standard post-treatment care.

#### Porter et al. [28]

Porter, McConnell [28] developed music therapy sessions for hospice patients in Northern Ireland with an Eastern Cooperative Oncology Group (ECOG) performance of 2 or lower. The intervention group received a total of six 45-min individual music therapy sessions, twice a week. The control group underwent usual cancer care. A trained and registered music therapist provided the program using an interactive approach. Patients could participate by singing or listening to known music, but they also got the opportunity to create something of their own, e.g., a melody, song, rhythm, or instrumental piece. The music therapist supported the patients in the creative process.

#### Radl et al. [24]

Self-Book art therapy was offered by Radl and Vita [24] to female cancer patients undergoing active oncological treatment in a major hospital in Philadelphia, USA. Both the intervention group and the control group had access to all available complementary (psychological) therapies, but only the intervention group created a Self-Book. The participants worked with an art therapist individually in six sessions (“agreements”) of about 50 min. The purpose of the art therapy was to create a self-reflective book to express one’s feelings and experiences. During the first five sessions, the patients were instructed to fill the pages of their book with creative artwork related to a given subject (safe place, supports, strength and virtues, wishes for loved ones, wishes for oneself). In the final session, the patients were encouraged to decorate the cover of the book.

### Findings: effect on outcome measures

#### Anxiety

Out of the four studies measuring anxiety, two found a significant improvement. De Feudis and Graziano [26] reported a significant reduction in anxiety scores in the intervention group, with the score decreasing from 44.3 to 37.1 (*p* = 0.002), while the anxiety scores in the control group did not significantly change. However, the study did not find a significant difference in anxiety scores between the two groups. In the study of Jang and Kang [23], anxiety scores were significantly improved compared to the control group (*p* < 0.001). Geue and Richter [27] did not find any significant differences, neither within the invention group nor between the intervention group and control group. Bozcuk and Ozcan [25] compared anxiety scores among two intervention groups and one control group and found that anxiety scores did not differ significantly between the group.

#### Depression

Three of the seven studies compared depression scores between intervention and control groups. Jang and Kang [23] and Bozcuk and Ozcan [25] found the depression scores in the intervention group to significantly improved compared to the control group (*p* < 0.001 and *p* = 0.001 respectively). Geue and Richter [27] found neither significant improvement in depression scores within the groups nor between the groups.

#### Quality of life

Six studies reported on QoL or QoL related scales, such as well-being, of which four found an improvement in these outcome variables. Bozcuk and Ozcan [25] reported a significant difference in QoL between the intervention groups and the control group (*p* = 0.001). In addition, as expected through the regression to the mean principle, patients with lower QoL appeared to take the greatest advantage from painting art therapy program. All participants declared they enjoyed taking part in painting art therapy program. The intervention was also found to be feasible during chemotherapy sessions. Jang and Kang [23] also reported improvement in quality of life, with the global health status/QoL score increasing from 26.4 to 81.3 (*p* < 0.001). Significant beneficial effects on functional scales, physical symptoms, and financial difficulties were also noted. None of these changes were found in the control group. Additionally, Jalambadani and Borji [22] showed statistically significant decreases in symptoms of distress in the intervention group compared to the waiting list control group. The scores of physical health, psychological symptoms, social relationships, and environmental factors were improved significantly, as well as quality of life behavior. Lastly, De Feudis and Graziano [26] reported that 89.3% of the participating patients considered the art therapy program beneficial to their well-being.

Radl and Vita [24] documented no statistically significant differences between the Self-Book therapy intervention group and the control group for the primary outcome (emotional distress) or the secondary outcome (psychological well-being). However, they did find significant improvement in the spiritual well-being of the patients taking part in the Self-Book art therapy program. Also in the study of Porter and McConnell [28] changes in McGill Quality of Life questionnaire (MQoL) scores, as well as in physical symptoms and psychological and existential well-being, from baseline to the first assessment (week 1) were not statistically different between the intervention group and the control group [28].

#### Summary results

In conclusion, of the seven studies, four identified significant results regarding anxiety, depression, or QoL [22, 23, 25, 26]. Of the four studies that studied anxiety, half found significant improvements in anxiety scores, the other half did not [23, 26]. Regarding depression, two studies found significant improvement in depression scores and one did not [23, 25]. Four out of six studies regarding QoL showed significant improvement in QoL after the art therapy intervention [22, 23, 25]. Hence, three studies did not identify any significant results regarding anxiety, depression, or QoL [24, 27, 28]. Nevertheless, all participants considered the experience valuable to their well-being, what came up anecdotally as well as through questionnaires after completion of the intervention. An overview of the results of all studies can be found in Table 2.

**Table 2:** Study results

Reference	Study design	Participants				Intervention			Outcome measures	Main findings
		Diagnosis	*N*^1^	F:M^2^	IG:CG^3^	Methods	Duration	Instructor		
Bozcuk et al. [25]	3-group comparative study	Adult cancer patients receiving chemotherapy	97	54:43	65:32	Painting art therapy program (PATP): introduction about technique and materials, making watercolor paintings, elaborating about the meaning and subject of the paintings	6 weeks	Professional painting artist	EORTC-QLQ-C30 questionnaire; Hospital Anxiety and Depression Scale (HADS)	- Significant improved QoL and decreased depression in cancer patients who received PATP - All participants enjoyed the intervention to some extent - PATP may be of more benefit to patients who are relatively in more need of help - PATP is feasible during chemotherapy sessions
De Feudis et al. [26]	Non-randomized pre-post study design	Adult cancer patients	115	88:27	59:56	The production of spontaneous artwork; provoking individual self-reflections connected to the art work; shared meaning—making within the group	4 months; each participant took part in one session (90 min)	Therapist with expertise in art therapy	State-Trait Anxiety Inventory-Form (STAI-Y); Edmonton Symptom Assessment Scale-Revised (ESAS-R); two open-ended questions about satisfaction with the intervention	- Significant reduction in anxiety and psychosomatic distress symptoms (drowsiness and fatigue) were found in the IG compared to the CG - Most participants perceived art therapy as having a positive influence on their well-being - Intervention format was considered appropriate by other staff members
Geue et al. [27]	Prospective intervention study	Adult cancer patients who had just finished acute treatment	183	94:89	54:129	Structuring materials and practicing experimental drawing; introducing watercolors; creating an individual book	22 weekly sessions of 90 min	Artist with psycho-oncological training	Hospital Anxiety and Depression Scale (HADS); Questionnaire on Coping with Illness (FKV); Perceived Adjustment to Chronic Illness Scale (PACIS)	- No changes in depression scores were found for the IG - Anxiety scores decreased in a pre-post comparison, but there were no significant differences with the CG -Subjective experiences were positive throughout
Jalambadani et al. [22]	Semi-experimental study	Women with breast cancer, any stage	124	124:0	Unclear	The 8-week MBAT program of Monti et al. (2006)	12 weeks; once a week; 90 min	Artist with psycho-oncological training	WHO Quality-of-Life (WHOQOL)-BREF questionnaire	- Patients in the IG showed significant decreases in symptoms of distress compared to the CG - Support for the hypothesis that MBAT intervention can help decrease distress levels and improve QoL
Jang et al. [23]	Randomized controlled trial	Women with breast cancer stage 0-III	24	24:0	12:12	Korean mindfulness-based stress reduction’s (K-MBSR) psychological intervention combined with the 8-week MBAT program of Monti et al. (2006)	12 sessions; 45 min	Qualified art therapist	Personality Assessment Inventory (PAI); EORTC-QLQ-C30 questionnaire	- Compared to the CG, patients in the IG reported significantly decreased depressive symptoms after treatment - Compared to the CG, patients in the IG reported significantly decreased anxiety after treatment - Global health status in the IG was increased after the treatment period
Porter et al. [28]	Randomized controlled trial	Oncology hospice inpatients	51	36:15	25:26	Singing, playing, listening to known music; creating a melody, rhythm, song, or instrumental piece	Up to 6 individual sessions over 3 weeks	Trained and registered music therapist	McGill Quality of Life Questionnaire	- As expected, the change from baseline 1 was not significantly different between IG and CG - Notable improvement in existential well-being in IG compared to IG - Notable disimprovement in physical well-being in CG compared to IG
Radl et al. [24]	Randomized controlled trial	Female cancer patients	60	60:0	30:30	Creation of a journal-style, self-reflective visual book	6 sessions; 50 min	Art therapist	Distress Thermometer (DT); FACIT-Sp; Patient-Reported Outcomes Measurement System Brief Psychological Well-being test (PROMIS)	- No statistically significant differences between Self-Book art therapy in IG and CG for the primary outcome (emotional distress) or the secondary outcome (psychological well-being) - Statistically significant increase in participant’s spiritual well-being compared to the CG - Greater effects in younger participants

^1-4^*N* number of patients; *F:M* female to male ratio; *IG:CG* intervention group to control group ratio; *NA* not applicable

## Discussion

### Main findings

In this systematic review, we found some positive effects of art making on anxiety, depression, and QoL in adults with cancer. Four out of seven included studies described these beneficial effects. All studies reported that participants considered the experience valuable to their well-being.

### Interpretations

These results partially support the findings of previous non-controlled studies on the effectiveness of art therapy on psychological outcomes in cancer care [30–33]. This is somewhat encouraging, because novel, evidence-based interventions to improve psychological outcomes for cancer patients are urgently needed, especially in view of the increased life expectancy of this patient population [34], which prolongs the period of being ill. Whereas QoL is considered of main importance by cancer patients [35], psychological needs are still unrecognized and undertreated [36].

In our review, we only considered interventions involving active art making by the patients. Clearly, many other forms of art therapy interventions for cancer patients exist, from purely passive appreciation of art to more interactive forms such as co-creation, in which an artist creates art while making use of the patient’s narrative [37]. Active creative work is likely to differ in its mental impact from passive art consumption and is therefore best investigated separately.

### Strengths and limitations

#### Strengths

For this review, we did not only search the commonly used database PubMed/MEDLINE but also EMBASE and PsycINFO, providing an overview of the literature that is as complete as possible. Next, we used a stringent definition of art therapy, focusing on art making in the presence of an artist or art therapist only. In this way, the studies were highly comparable. We excluded all studies that did not use a control group, which increased the validity of our results. Finally, the included studies were from countries across the world, enhancing the generalizability of the results.

#### Limitations

Despite the strengths of this review, our findings need to be interpreted with caution. The included articles showed several methodological shortcomings. First, three out of seven studies were not randomized, which may have led to selection bias [25–27]. Randomization of the participants was only attempted in the RCTs, because others were afraid that randomization might decrease the willingness of patients to participate. Second, it is not entirely certain that all the effects found in the non-RCTs are due to the art therapy interventions, because the included studies did not address controlling for confounders. For instance, it was often not clear what the cancer characteristics, such as metastasized or non- metastasized, of the patients were. Third, there was a remarkable imbalance in participation between men and women, as three out of seven studies did not include male patients at all. Tavani already addresses the low number of male art therapists [38], but only few studies have elaborated why men are less likely to participate in art-making programs. This should be investigated further in order to make art therapy suitable for a general population. Finally, the widely varying cultural settings of the included studies are likely to have contributed to the heterogeneity of the studies.

### Clinical implications and future research

Our findings are supportive for further development of art-making approaches in cancer care. To improve the clinical evaluation of these approaches, the methodological shortcomings, e.g., the lack of randomization, need to be addressed. Outcomes of this review suggest that more randomized controlled trials with larger sample sizes are needed to establish the evidence of art therapy’s effectiveness for adults with cancer. We also recommend developing a protocol to standardize art therapy interventions that are also feasible for clinical practice and for continuation at home.

## Conclusion

In conclusion, art therapy involving a professional art therapist or artist and active art-making of the patients can possibly have a positive effect on anxiety, depression, and QoL in adults with cancer. However, further research with stringent definitions of art therapy as an intervention and appropriate randomized designs are urgently needed.

## Supplementary Information

ESM 1 (DOCX 29 kb)

ESM 2 (PDF 152 kb)
