# Supplementary material for: The effects of art therapy on anxiety, depression, and quality of life in adults with cancer: a systematic literature review
Source: Support Care Cancer. 2020 Nov 13;29(5):2289–98. doi: 10.1007/s00520-020-05869-0 (PMC7981299; doi:10.1007/s00520-020-05869-0)
Supplement: Supplementary file 1 — (DOCX 29 kb) [file 520_2020_5869_MOESM1_ESM.docx]

## Appendix A: Search strategy

### PubMed/MEDLINE

| (art therapy [MeSH] OR art therap* [tiab] OR art program [tiab] OR art-making [tiab] OR art making [tiab] OR performative arts [tiab] OR artwork [tiab] OR music therapy [MeSH])  AND  (neoplasms [MeSH] OR neoplasm* [tiab] OR neoplasia*[tiab] OR cancer*[tiab] OR oncolog*[tiab] OR tumour* [tiab] OR carcinoma* [tiab] OR malignan*[tiab]) |
| --- |

### PsycINFO

| (art therapy OR art therapist OR art program OR art-making OR performative arts OR music therapy)  AND  (neoplasms OR cancer OR oncology) |
| --- |

### EMBASE

| (art therapy/ OR (art-therap* OR art-making OR artwork OR music making).ti,ab,kw.)  AND  (neoplasm/ OR (neoplasm* OR cancer* OR oncolog* OR tumour* OR malignan*).ti,ab,kw.) |
| --- |

## Appendix B: Results of critical appraisal

### JBI Critical Appraisal Checklist for Quasi-Experimental Studies (non-randomised experimental studies)

#### Bozcuk, Ozcan ^28^

| 1 | Is it clear in the study what is the ‘cause’ and what is the ‘effect’ (i.e. there is no confusion about which variable comes first? | Yes, the cause is the watercolour art therapy intervention and the effects are changes in QoL, anxiety and depression |
| --- | --- | --- |
| 2 | Were the participants included in any comparisons similar? | No, the control group consists of patients who declined joining the treatment |
| 3 | Were the participants included in any comparisons receiving similar treatment/care, other than the exposure or intervention of interest? | Unclear, no explanation about treatment/care in the control group is given |
| 4 | Was there a control group? | Yes |
| 5 | Were there multiple measurements of the outcome both pre and post the intervention/exposure? | Yes |
| 6 | Was follow up complete and if not, were differences between groups in terms of their follow up adequately described and analysed? | Unclear, nowhere is stated of the follow-up was complete or not |
| 7 | Were the outcomes of participants included in any comparisons measured in the same way? | Yes |
| 8 | Were outcomes measured in a reliable way? | Yes, the EORTC QLQ-C30 and HADS |
| 9 | Was appropriate statistical analysis used? | Yes, ANOVA |

#### De Feudis, Graziano ^29^

| 1 | Is it clear in the study what is the ‘cause’ and what is the ‘effect’ (i.e. there is no confusion about which variable comes first? | Yes, the cause is the art therapy intervention and the effects are anxiety and distress |
| --- | --- | --- |
| 2 | Were the participants included in any comparisons similar? | Yes, patients recruited using the inclusion criteria were divided into two groups |
| 3 | Were the participants included in any comparisons receiving similar treatment/care, other than the exposure or intervention of interest? | No, the control group received usual care; not a comparable psychological intervention |
| 4 | Was there a control group? | Yes |
| 5 | Were there multiple measurements of the outcome both pre and post the intervention/exposure? | Yes, it is a pre-post-test design |
| 6 | Was follow up complete and if not, were differences between groups in terms of their follow up adequately described and analysed? | Yes, differences are displayed clearly in the diagram |
| 7 | Were the outcomes of participants included in any comparisons measured in the same way? | Yes |
| 8 | Were outcomes measured in a reliable way? | Yes, using the STAI-Y and ESAS-R |
| 9 | Was appropriate statistical analysis used? | Yes, ANOVA |

#### Geue, Richter ^30^

| 1 | Is it clear in the study what is the ‘cause’ and what is the ‘effect’ (i.e. there is no confusion about which variable comes first? | Yes, the cause is the art therapy intervention and the effects are changes in psychological distress |
| --- | --- | --- |
| 2 | Were the participants included in any comparisons similar? | No, significant differences and selection bias |
| 3 | Were the participants included in any comparisons receiving similar treatment/care, other than the exposure or intervention of interest? | Unclear, no explanation about treatment/care in the control group is given |
| 4 | Was there a control group? | Yes |
| 5 | Were there multiple measurements of the outcome both pre and post the intervention/exposure? | Yes, there were three points of measurement |
| 6 | Was follow up complete and if not, were differences between groups in terms of their follow up adequately described and analysed? | Yes, the loss-to-follow-up was described clearly |
| 7 | Were the outcomes of participants included in any comparisons measured in the same way? | Yes |
| 8 | Were outcomes measured in a reliable way? | Yes, using the HADS, FKV, PACIS |
| 9 | Was appropriate statistical analysis used? | Yes, ANOVA |

### JBI Critical Appraisal Checklist for Randomized Controlled Trials

#### Jalambadani and Borji ^25^

| 1 | Was true randomization used for assignment of participants to treatment groups? | Yes |
| --- | --- | --- |
| 2 | Was allocation to treatment groups concealed? | Unclear |
| 3 | Were treatment groups similar at baseline? | Yes, the groups were randomly assigned |
| 4 | Were participants blind to treatment assignment? | Yes, the control group was assigned to a waiting list |
| 5 | Were those delivering treatment blind to treatment assignment? | Not applicable, delivering art therapy cannot be blinded |
| 6 | Were outcomes assessors blind to treatment assignment? | Unclear |
| 7 | Were treatment groups treated identically other than the intervention of interest? | Yes, the control group received usual care |
| 8 | Was follow up complete and if not, were differences between groups in terms of their follow up adequately described and analysed? | No, the loss to follow-up is not well described |
| 9 | Were participants analysed in the groups to which they were randomized? | Yes |
| 10 | Were outcomes measured in the same way for treatment groups? | Yes |
| 11 | Were outcomes measured in a reliable way? | Yes, using the WHOQOL-BREF questionnaire |
| 12 | Was appropriate statistical analysis used? | Yes |
| 13 | Was the trial design appropriate, and any deviations from the standard RCT design (individual randomization, parallel groups) accounted for in the conduct and analysis of the trial? | Yes |

#### Jang, Kang ^26^

| 1 | Was true randomization used for assignment of participants to treatment groups? | Yes |
| --- | --- | --- |
| 2 | Was allocation to treatment groups concealed? | Unclear |
| 3 | Were treatment groups similar at baseline? | No, the values of depression and anxiety are lower in the control group |
| 4 | Were participants blind to treatment assignment? | Yes, the control group was assigned to a waiting list |
| 5 | Were those delivering treatment blind to treatment assignment? | Not applicable, delivering art therapy cannot be blinded |
| 6 | Were outcomes assessors blind to treatment assignment? | Unclear |
| 7 | Were treatment groups treated identically other than the intervention of interest? | Yes, the control group was on a waiting list |
| 8 | Was follow up complete and if not, were differences between groups in terms of their follow up adequately described and analysed? | Yes, the follow-up and analysis are showed in the flowchart |
| 9 | Were participants analysed in the groups to which they were randomized? | Yes, following the intention to treat principle |
| 10 | Were outcomes measured in the same way for treatment groups? | Yes |
| 11 | Were outcomes measured in a reliable way? | Yes, using the PAI and EORTC QLQ-C30 |
| 12 | Was appropriate statistical analysis used? | Yes, following the intention to treat principle |
| 13 | Was the trial design appropriate, and any deviations from the standard RCT design (individual randomization, parallel groups) accounted for in the conduct and analysis of the trial? | Yes |

#### Porter, McConnell ^31^

| 1 | Was true randomization used for assignment of participants to treatment groups? | Yes |
| --- | --- | --- |
| 2 | Was allocation to treatment groups concealed? | Yes, via an independent statistician |
| 3 | Were treatment groups similar at baseline? | Yes |
| 4 | Were participants blind to treatment assignment? | Unclear |
| 5 | Were those delivering treatment blind to treatment assignment? | Not applicable, delivering art therapy cannot be blinded |
| 6 | Were outcomes assessors blind to treatment assignment? | Yes, the treatment allocation was forwarded directly to the music therapist by the clinical investigator |
| 7 | Were treatment groups treated identically other than the intervention of interest? | Yes, the control group received usual  care |
| 8 | Was follow up complete and if not, were differences between groups in terms of their follow up adequately described and analysed? | Yes, the loss to follow-up was described clearly |
| 9 | Were participants analysed in the groups to which they were randomized? | No, a lot of patients lost to follow up |
| 10 | Were outcomes measured in the same way for treatment groups? | Yes |
| 11 | Were outcomes measured in a reliable way? | Yes, using the McGill Quality of Life questionnaire |
| 12 | Was appropriate statistical analysis used? | Unclear |
| 13 | Was the trial design appropriate, and any deviations from the standard RCT design (individual randomization, parallel groups) accounted for in the conduct and analysis of the trial? | Yes |

#### Radl, Vita ^27^

| 1 | Was true randomization used for assignment of participants to treatment groups? | Yes |
| --- | --- | --- |
| 2 | Was allocation to treatment groups concealed? | Yes, permuted blocked randomization schedule |
| 3 | Were treatment groups similar at baseline? | Yes |
| 4 | Were participants blind to treatment assignment? | Yes |
| 5 | Were those delivering treatment blind to treatment assignment? | Not applicable, delivering art therapy cannot be blinded |
| 6 | Were outcomes assessors blind to treatment assignment? | No, due to limited funding there was no blinding of outcome assessors |
| 7 | Were treatment groups treated identically other than the intervention of interest? | Yes, the only difference was the making of a Self-Book |
| 8 | Was follow up complete and if not, were differences between groups in terms of their follow up adequately described and analysed? | Yes, the loss to follow-up is showed in the diagram |
| 9 | Were participants analysed in the groups to which they were randomized? | Yes |
| 10 | Were outcomes measured in the same way for treatment groups? | Yes |
| 11 | Were outcomes measured in a reliable way? | No, they doubt the reliability of the FACIT-Sp and the DT themselves |
| 12 | Was appropriate statistical analysis used? | Yes |
| 13 | Was the trial design appropriate, and any deviations from the standard RCT design (individual randomization, parallel groups) accounted for in the conduct and analysis of the trial? | Yes |
